# Cardiac lead perforation: Mechanisms, detection, and therapeutic approaches

**DOI:** 10.1016/j.hroo.2025.08.008

**Published:** 2025-08-11

**Authors:** Ameer Awashra, Mohammed AbuBaha, Hossam Salameh, Hammam Jallad, Aya Milhem, Anwar Zahran, Aseel Badwan, Fathi Milhem, Abdalhakim Shubietah

**Affiliations:** 1 Department of Medicine, An Najah National University, Nablus, Palestine; 2 School of Medicine, The University of Jordan, Amman, Jordan; 3 Department of Medicine, Advocate Illinois Masonic Medical Center, Illinois

**Keywords:** Cardiac lead perforation, Pacemaker lead perforation, Lead dislodgement, Device-related complications, Transvenous pacing, Surgical revision, Lead extraction

## Abstract

Cardiac lead perforation is an infrequent but serious complication of cardiac implantable electronic devices, with possibly lethal consequences if left untreated. Because of its very varied clinical appearance and lack of established criteria, perforation still presents diagnostic and therapeutic hurdles despite continuous improvements in lead design and insertion procedures. This review summarizes current research on the processes, risk factors, and clinical range of lead perforation. Thin myocardial walls, especially at the right ventricular apex; active-fixation leads; and operator-dependent elements, such as lead placement and torque, are important anatomic and procedural contributions. Steroid usage, low body mass index, and female sex all increase risk. A strong index of suspicion is often required for prompt diagnosis because presentations vary from asymptomatic instances to severe tamponade. Device interrogation must be integrated with imaging modalities, particularly computed tomography and echocardiography, which each have unique benefits based on the clinical setting, to achieve accurate detection. From cautious observation in stable patients to immediate percutaneous or surgical intervention in unstable or worsening situations, management approaches are highly customized. New technologies, including subcutaneous implantable cardioverter-defibrillators and leadless pacemakers, have the potential to lower the incidence of perforations, but they also present new difficulties that need to be further investigated. Importantly, the lack of consistent diagnostic standards hinders study/ comparability and postpones the creation of guidelines. Standardizing diagnostic criteria, establishing multicenter prospective registries, developing machine learning-based risk stratification tools, and conducting clinical trials to inform the treatment of asymptomatic or delayed perforations are among the top goals for the future. To improve patient outcomes and advance clinical practice, international cooperation and uniform reporting standards are crucial.

## Graphical abstract

**Figure:**
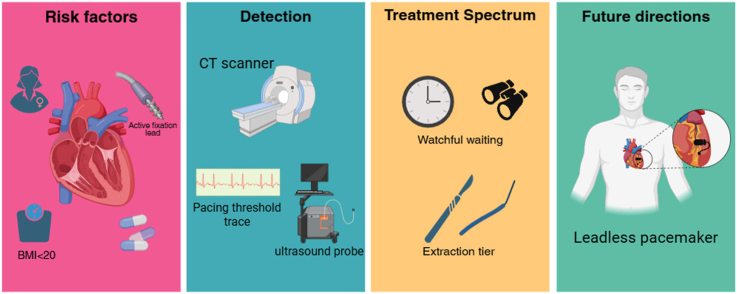

**Key Findings**

▪ Cardiac lead perforation is a rare but serious complication of cardiac implantable electronic device implantation, influenced by factors such as lead type, implant site, age, sex, and body mass index.
▪ Diagnosis requires high clinical suspicion; computed tomography imaging provides the best accuracy for identifying perforation and lead position.
▪ Management depends on patient stability, ranging from monitoring in asymptomatic cases to urgent lead extraction in unstable patients.
▪ Active fixation leads and apical placement increase perforation risk, whereas septal placement and passive leads are safer for high-risk patients.
▪ There is a need for standardized diagnostic criteria, multicenter registries, and machine learning models to improve detection and guide management.

Since their debut, cardiac implanted electronic devices, such as implantable cardioverter-defibrillators (ICDs), permanent pacemakers, and cardiac resynchronization therapy systems, have revolutionized the treatment of heart failure and cardiac rhythm abnormalities.^1^ These devices have seen substantial improvement in terms of usefulness, safety, and miniaturization since they were first created in the middle of the 20th century.^2^ With more than 1.5 million new implantations performed worldwide each year, these devices have become crucial therapeutic instruments.^3^

Even with advancements in technology, lead-related problems are still common; cardiac lead perforation is an uncommon but potentially fatal occurrence.^4^ Despite advancements in design and implantation methods, this complication, which was first identified in the early days of cardiac pacing, continues to pose diagnostic and treatment difficulties.^5^ When an implanted lead breaks through the myocardium and enters the pericardial space or nearby tissues, it is known as a cardiac lead perforation.^6^ Temporally, this issue is categorized as delayed/late (>1 month), subacute (24 hours to 1 month), or acute (≤24 hours after implantation).^6^ Because it influences the clinical presentation, diagnostic process, and therapeutic strategy, this temporal categorization is crucial.^7^

Pacemaker and ICD leads have recorded incidences ranging from 0.1% to 0.8%^8^ and 0.6% to 5.2%,^8^ respectively. The higher incidence rates for ICD leads are ascribed to their larger diameter and greater stiffness; cardiac resynchronization therapy systems could be involved, albeit there is currently little information available on these leads because of variations in lead location and setup.^9^

Pneumothorax, hemothorax, pericardial effusion, cardiac tamponade, and device malfunction are among the clinically significant outcomes that go beyond the acute procedural problems.^10^ Diagnostic delays may result from these problems’ presentations, which might mirror those of other illnesses and vary from asymptomatic patients to life-threatening situations.^11^ Depending on time, clinical presentation, hemodynamic stability, lead characteristics, and institutional competence, management strategies might vary from conservative monitoring to percutaneous lead extraction or surgical intervention.^10^

A mix of imaging modalities, such as computed tomography, transthoracic or transesophageal echocardiography, and chest radiography, is necessary for an accurate diagnosis.^12^ Depending on the clinical situation, each modality has a different diagnostic value.^4^

To raise clinician awareness, improve detection, optimize care, and lower related morbidity and mortality, this review summarizes the state of knowledge about cardiac lead perforation, with a focus on causes, risk factors, diagnostic techniques, and therapeutic measures.^7^

## Anatomic considerations and risk assessment

### Cardiac anatomy relevant to lead placement

The right ventricle (RV), which has 3 separate compartments, inlet, outflow, and apical, is the main chamber used for ventricular lead implantation. Every location has distinct features that influence the stability of lead and the likelihood of perforation.^13^

From the tricuspid valve to the papillary muscle insertions, the RV inlet is distinguished by conspicuous trabeculations that provide stability for passive-fixation leads but may make lead navigation more difficult.^13^ Because it is linked to more physiological activation patterns than standard apical pacing, the RV outflow tract, which is situated between the supraventricular crest and the pulmonary valve, is being used more and more.^14^

Owing to its much thinner myocardial walls (typically <1–2 mm) than other segments, the RV apex, traditionally the most popular location for ventricular lead placement, has a much higher risk of perforation.^13^ Research shows that RV apical leads had a 3.4-fold increased risk of perforation compared with nonapical placements.^15^

**Figure 1:**
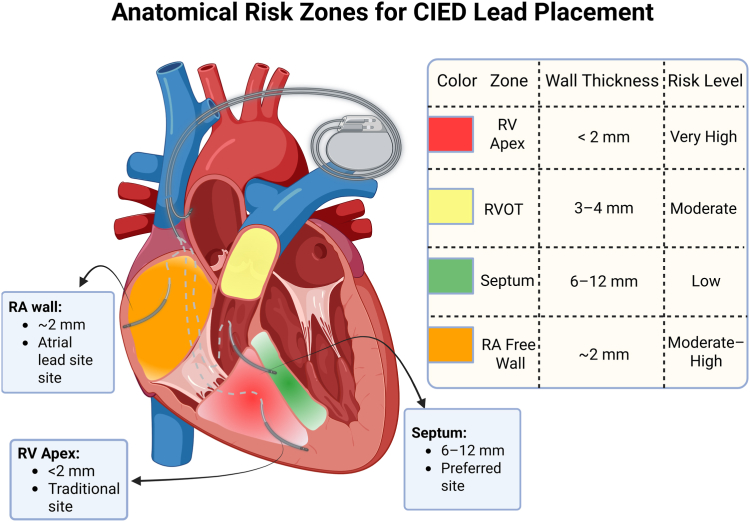
Right-heart cross-section with color-coded perforation-risk overlays. *Red* marks the RV apex (<2 mm wall, very high risk); *yellow* marks the RV outflow tract (3–4 mm, moderate risk); *orange* marks the RA free wall (∼2 mm, moderate to high risk); *green* marks the IVS (6–12 mm, low risk). Icons indicate typical CIED lead positions: traditional RV apical, preferred midseptal, and atrial appendage sites. CIED = cardiac implantable electronic device; IVS = interventricular septum; RA = right atrium; RV = right ventricle; RVOT = right ventricular outflow tract.

A popular target for modern lead implantation is the interventricular septum (IVS), especially the midsection close to the moderator band. Greater myocardial wall thickness (usually 6–12 mm), a lower risk of perforation, and the possibility of more physiological ventricular activation patterns are all benefits of the septum.^13^ In addition to providing mechanical support, structures such as the moderator band and septomarginal trabecula may aid in precisely directing septal lead attachment (shown in Figure 1).

There are special concerns for lead insertion in the right atrium (RA). Because pectinate muscles provide stability, the RA appendage has historically been favored. The free wall is more vulnerable to perforation, and the RA wall is typically thinner than the RV (∼2 mm).^16^

### Patient-related anatomic risk factors

Perforation risk is greatly influenced by a number of patient-specific variables. These include both inherent heart traits and more general physiological differences across groups. Progressive myocardial fibrosis, fatty infiltration, and thinning with age are examples of age-related cardiac abnormalities that might modify tissue mechanical characteristics and perhaps make a person more vulnerable to perforation.^17^

Differences by gender have also been reported. Owing to their smaller heart chambers and thinner myocardial walls, female patients have a much greater incidence of perforations—2–3 times higher.^18^ Another crucial factor to consider is body habitus. Reduced myocardial wall thickness and lower heart dimensions are probably the causes of the high correlation between low body mass index (BMI) (<20 kg/m^2^) and increased perforation risk.^19^

Congenital (such as Ebstein’s abnormality) and acquired structural anomalies (such as ventricular aneurysms) provide special anatomic obstacles and may need altered lead placement techniques. Lead trajectory planning may also be affected by disorders such as hypertrophic cardiomyopathy or postsurgical remodeling, which may skew typical anatomic markers.^20^

### Preprocedural anatomic assessment

Planning structural and electrophysiological cardiac treatments to improve safety and efficacy requires preprocedural anatomic examination.^21^ This entails choosing the right imaging modalities according to clinical requirements: computed tomography (CT) for detailed anatomic visualization and vascular access planning^21^; cardiac MRI for tissue characterization, particularly in detecting fibrosis or thinning^22^; and transthoracic echocardiography (TTE) for baseline heart function.^21^ For valvular evaluation, transesophageal or 3D echocardiography may provide more information.^23^ To guide the procedural approach, it is necessary to identify key anatomic hazards, such as myocardial infarction at proposed device locations, myocardial thinning (<1 mm), aneurysms, calcification, complicated valve architecture, thrombi, tumors, or problematic vascular access.^21^ To prevent difficulties, precise lead or device trajectory planning is necessary, and 3D reconstruction for ideal placement is made possible by CT or cardiac MRI.^22^ When doing cardiac implanted electronic device (CIED) operations, it is also important to assess the patient’s cardiac rhythm and device history.

## Mechanisms of cardiac lead perforation

Owing to the nature of the ever-changing anatomy of the heart chambers with each cardiac cycle and respiratory motion, the interaction between cardiac leads and the myocardium is complex.^24^

Cardiac lead perforations can be classified according to the time it takes to develop signs of perforation after surgery, with acute perforations developing within the first 24 hours, early perforations developing within 30 days, and delayed perforations developing after 30 days, respectively, according to Kumar et al.^19^

Cameron et al^24^ attributed cardiac wall perforation to the material of the used leads, given that there have been experiments where silicone rubber leads, instead of polyurethane-coated leads, yielded less stiff leads. However, the use of this material resulted in thinner leads with a higher penetration pressure owing to the force exerted on the wall by the thinner lead, which is further supported by Gasser et al^25^ in their experiment where lower pressure points resulted from a wider diameter used.

Tissue-related factors include myocardial tissue stiffness and elasticity, which, according to Gasser et al,^25^ are not the same throughout chambers and even within the chamber itself, described as heterogeneity of the myocardial tissue.

Although RA wall perforation still occur a lot of the times, recent data on almost 2400 patients show that RV anterior wall perforation was more common than the RA wall perforation, which is surprising because the RA is thinner,^26^ explained by the fact that the anterior wall is thin and can be easily perforated, in contrast with the septum, which can be 2 times in thickness.^27^

Perforation of the cardiac lead or migration into the left side of the heart is less common. It can rarely happen owing to various etiologies such as an atrial septal defect^28^ and a patent foramen ovale^29^ or even through the IVS.^30^ Even though these complications seem serious, according to the literature, they can be asymptomatic for a long time and might be discovered incidentally.^28^

With the increased frequency of surgeries using the coronary sinus as an access to left ventricular lead insertion, the rates of coronary sinus perforation/dissection have been reported to be as high as 2%^29^ and even extend to cause pericarditis and/or cardiac tamponade.^30^

**Figure 2:**
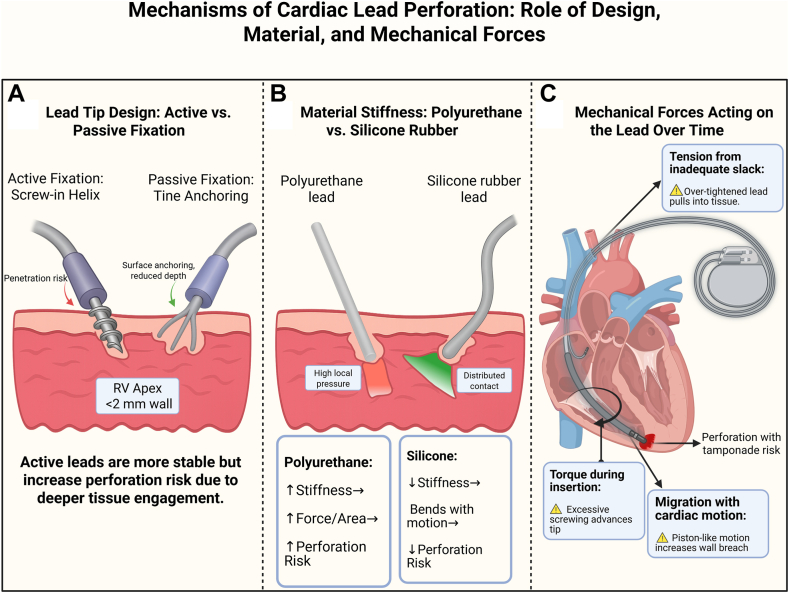
Mechanisms of cardiac lead perforation. **A:** Comparison between active screw-in and passive tine lead designs and their interaction with endocardial tissue. **B:** Effect of material stiffness—polyurethane exerts focal pressure, whereas silicone distributes force more safely. **C:** Torque, slack, and postprocedural migration contribute to progressive tissue damage and perforation. RV = right ventricle.

As stated earlier, lead design and fixation types play into the role of cardiac perforation. Two types of lead fixation exist: active fixation, where the lead is inserted or “screwed into” the endocardium, and passive fixation, where the lead is passively fixed in the RV’s apex, where trabeculations function as an anchoring site for the lead.^19^ It seems that the active-fixation type leads demonstrate higher rates of cardiac perforation.^26^ Most cardiac lead perforations occur at the cardiac apex because it is a common site of lead insertion^31^ (shown in Figure 2).

## Risk factors for lead perforation

Risk factors for lead perforation can vary and have both intrinsic and extrinsic causes, such as myocardial wall thickness and physician expertise, respectively. Sterlinski et al,^32^ in their retrospective analysis on 2247 leads in 1419 patients, have categorized factors that may be involved in the process of cardiac perforation into lead design, surgeon experience, and patient-specific factors.

The use of helical screw leads has been associated with a higher risk of perforation, which was consistent with the findings in Sterlinski’s study,^32^ which showed that all perforations that occurred were related to the use of retractable screw leads.^33^

Even in highly skilled and experienced physicians, certain errors can still occur, such as overtorquing and leaving an excessive loop, which is associated with an increased risk of cardiac perforations owing to the forward movement of the lead and the associated excessive tension applied.^31^^,^^32^

Various patient-specific factors are associated with an increased risk of cardiac perforations and were categorized by Mahapatra et al^33^ according to postimplant effusion where a univariate analysis on 4280 implanted permanent pacemakers showed 2 strongly associated predictors such as the concomitant use of temporary transvenous pacemaker (hazard ratio [HR] 3.2; 95% confidence interval [CI] 1.6–6.2; *P* = .001) or recent steroid use (HR 4.1; 95% CI 1.1–10; *P* = .003). Other patient-specific predictors were a BMI of less than 20 kg/m^2^ (HR 2.5) and old age (HR 1.4).^33^

Other procedural risk factors that are considered patient specific included temporary pacemaker wire use (HR 2.7; 95% CI 1.4–3.9; *P* = .01) and longer fluoroscopy times (HR 1.3).^33^ However, the only variable that was independently associated with a reduced risk of cardiac perforation was an RV systolic pressure of >35 mm Hg (HR 0.70; 95% CI 0.50–0.92; *P* = .02) according to Mahapatra et al.^33^

In a study conducted by Hsu et al^34^ of more than 440,000 first-time implantable cardioverter-defibrillator (ICD) patients, cardiac lead perforation occurred in just 0.14% of cases. Older age, being female, having heart failure, or receiving a more complex device increased the risk. In contrast, atrial fibrillation, diabetes, past bypass surgery, and having a more experienced implanter reduced the risk. Patients who experienced perforation were much more likely to have serious complications, have longer hospital stays, and even die during their hospital stay (shown in Table 1).

**Table 1:** Comparative summary of risk factors for cardiac lead perforation

Category	Risk factor	Impact on perforation risk
Anatomic location^13^	Right ventricular apex	High (thin myocardial wall <1 to 2 mm)
	Interventricular septum	Lower (thicker wall 6–12 mm)
Patient characteristics^18^^,^^19^	Female sex	Higher risk (2–3 times more frequent)
	Low BMI (<20 kg/m^2^)	Increased risk owing to a smaller heart size
Lead Type^35^^,^^36^	Active-fixation lead	Higher perforation incidence
	Passive-fixation lead	Lower risk in stable anatomy

BMI = body mass index.

## Clinical presentation and diagnosis

The clinical range of cardiac lead perforation is wide, spanning from life-threatening cardiac tamponade to asymptomatic electrical abnormalities. The degree of myocardial breach, lead type, anatomic location, and date of commencement are some of the variables that affect its clinical variability. High clinical suspicion and a methodical diagnostic approach are necessary for timely diagnosis, owing to the possibly subtle indications, to prevent intervention delays and minimize negative effects.^37^^,^^38^

### Symptomatology spectrum

Lead perforation symptoms may manifest in a wide range of ways. With 25%–70% of patients, chest discomfort is still the most often reported symptom. It is usually characterized as positional, pleuritic, and acute.^4^^,^^33^ Approximately 30% of patients have respiratory problems, including dyspnea, which may vary from minor discomfort during exercise to severe respiratory distress linked to pericardial effusion or tamponade.^15^ Both pacing failure and abrupt hemodynamic compromise from tamponade physiology may cause syncope and presyncope, especially in patients who are pacemaker dependent.^8^ Furthermore, diaphragmatic or intercostal muscle activation brought on by extracardiac stimulation via lead migration may clinically show up as hiccups or obvious twitches of the chest or abdominal wall.^39^^,^^40^

The symptom profile is also influenced by the time of the perforation. Acute perforations often manifest suddenly with symptoms including pericardial tamponade, hypotension, or acute chest discomfort, and they frequently happen intraoperatively or within 24 hours after device installation.^41^ Subacute perforations usually manifest as gradual, nonspecific symptoms that often mirror pulmonary or musculoskeletal disorders and develop over days to weeks after implantation.^41^^,^^42^ In contrast, delayed perforations, which are often discovered by chance during device interrogation or imaging tests performed for unrelated purposes, could not show clinical symptoms for weeks or even months after implantation.^43^

Crucially, up to 15%–30% of cardiac lead perforations could not cause any symptoms at all and can only be found by accident by imaging or regular device follow-up.^12^^,^^44^ During regular pacemaker or ICD interrogations, these quiet holes highlight the need to be alert when interpreting electrical anomalies.^45^

### Physical examination findings

Even though physical symptoms are sometimes vague, a careful examination might provide important hints that indicate a lead perforation. Hypotension, tachycardia, and raised jugular vein pressure are all signs of pericardial tamponade and may indicate hemodynamic instability.^46^ Pulsus paradoxus, which is characterized as an inspiratory decrease in systolic blood pressure of more than 10 mm Hg, is one of the most distinctive physical findings in this setting. It implies inadequate ventricular filling as a result of pericardial pressure.^47^

When a pericardial effusion is present, cardiac auscultation may show muffled heart sounds.^9^ Furthermore, a pericardial friction rub may sometimes be seen, signifying pericardial inflammation brought on by the displaced lead’s mechanical irritation.^48^ Rarely, pacing impulses may cause noticeable muscle twitches across the chest or upper abdomen, which would indicate the placement and stimulation of an extracardiac lead.^49^^,^^50^ Even though it is rare, subcutaneous emphysema may occur when a perforation is exacerbated by pneumothorax or pneumopericardium.^51^

### Device interrogation and electrical parameter assessment

The earliest and most impartial proof of lead perforation is often provided via device questioning. According to reports, 60%–70% of perforations increase pacing threshold, which is defined as more than a 2-fold increase from the postimplant baseline. This is usually caused by the decreased myocardial contact when the lead tip leaves the endocardial surface.^20^^,^^52^ In contrast, because of the increased impedance mismatch and nonmyocardial stimulation, partial perforation may paradoxically lead to lower thresholds.^53^

Abnormalities in sensing are also frequent; 40%–60% of patients have R-wave or P-wave amplitudes that are less than 50% of baseline values, which indicates less than ideal contact with cardiac tissue.^20^^,^^54^ Inappropriate pacing inhibition or the administration of needless treatments in defibrillator devices may be symptoms of oversensing of extracardiac myopotentials or diaphragmatic signals.^55^ Although less dependable, lead impedance may change based on the medium in which the lead tip is located. When fluid builds up, as in pericardial effusion, or when air pressure rises, as in pneumothorax or pneumopericardium, it usually falls.^21^^,^^48^^,^^56^ Advanced perforation may result in exit block or capture failure, which means that the lead has completely passed through the myocardial barrier even when there is no myocardial depolarization.^57^^,^^58^

### Imaging modalities

Given that no one imaging method is always conclusive, a multimodal imaging strategy is often required to confirm the diagnosis of lead perforation. Despite being readily accessible, chest radiography has a modest sensitivity (∼50%–60%) and may show indirect indicators such as aberrant lead curvature or extension outside of the cardiac profile.^59^, ^60^, ^61^

TTE is essential for quickly assessing heart chamber function and pericardial effusion. Acoustic shadowing often restricts accurate localization of the perforation site, despite having a sensitivity of approximately 70%–80% for seeing the actual lead tip.^62^, ^63^, ^64^ Widely regarded as the gold standard, chest CT has a diagnostic sensitivity of more than 95% and a specificity of more than 90%.^65^, ^66^, ^67^ Accurate lead location identification about the cardiac shape and surrounding thoracic structures is made possible by multiplanar reconstructions using CT.^5^ Intravenous contrast may improve the diagnosis of vascular damage or active extravasation in complicated situations, even though noncontrast CT is usually adequate.^68^

Although chest CT is highly accurate, some limitations should be accounted for, such as respiratory and cardiac contractile motion during imaging, which may overestimate the location, given that it has been recorded that cardiac motion varies between patients and could range from 1 mm to 15.2 mm, as evidenced by Wu et al.^69^

Transesophageal echocardiography, one of the other imaging methods, is especially helpful for identifying atrial lead perforations because of its proximity to cardiac structures and better resolution.^70^ When other modalities are unable to offer conclusive information and patient safety permits, cardiac magnetic resonance imaging (MRI) may be considered. Although traditionally contraindicated in patients with non-MRI-conditional leads, recent studies have reported the safe performance of MRI under controlled conditions in select cases. However, in the context of lead perforation, data are limited, and MRI is generally avoided owing to concerns over lead heating, dislodgment, or exacerbation of myocardial injury.^71^, ^72^, ^73^, ^74^, ^75^ For instance, Nazarian et al^74^ have demonstrated protocols for MRI in patients with pacemakers or defibrillators, but these typically excluded those with evidence of lead perforation (shown in Table 2).

**Table 2:** Diagnostic modalities for cardiac lead perforation

Modality	Sensitivity	Advantages	Limitations
Chest radiography^12^	50%–60%	Quick and accessible	Low sensitivity, indirect signs
Transthoracic echocardiography (TTE)^12^	70%–80%	Identifies effusion, bedside use	Poor visualization of lead tip
CT scan^12^	>95%	High resolution, multiplanar	Radiation exposure, not always immediate
Transesophageal echocardiography (TEE)^16^	High for atrial leads	High resolution near RA	Semi-invasive

CT = computed tomography; RA = right atrium.

### Diagnostic algorithm

**Figure 3:**
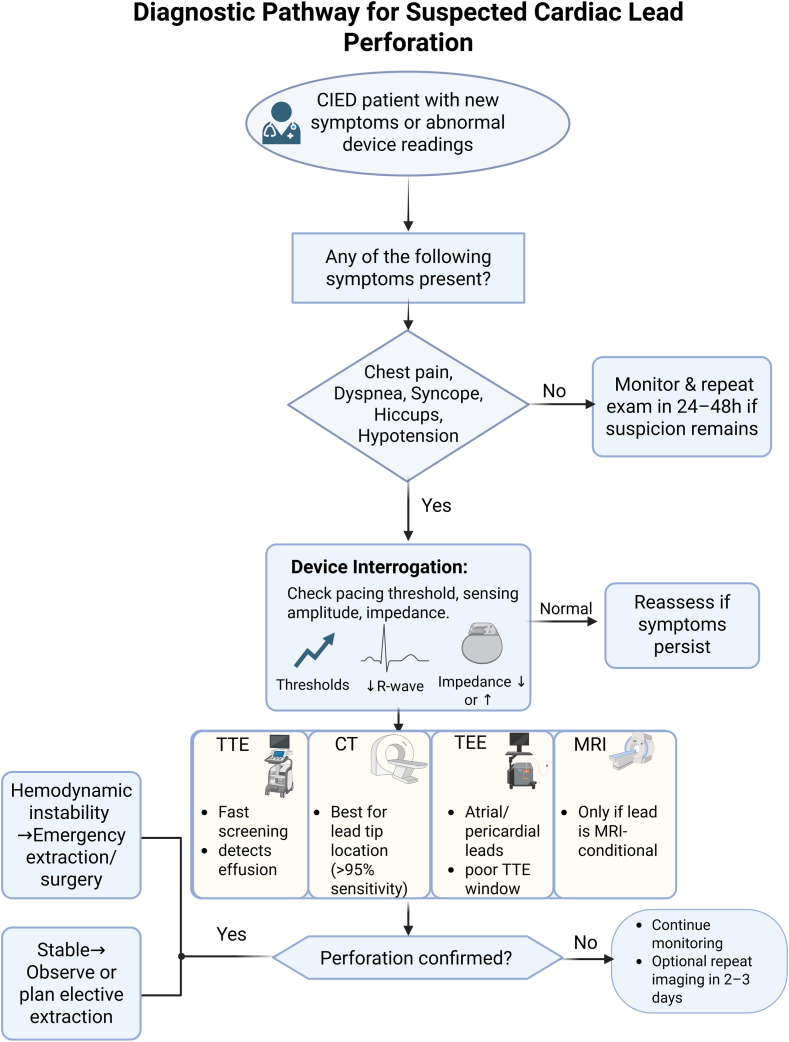
Diagnostic algorithm for suspected cardiac lead perforation, integrating symptom recognition, device interrogation, and multimodal imaging. Management depends on clinical stability and imaging confirmation. CT = computed tomography; MRI = magnetic resonance imaging; TEE = transesophageal echocardiography.

Clinical assessment, device interrogation, and imaging tests are all integrated in a step-by-step, tiered manner in an efficient diagnostic approach to suspected lead perforation. A comprehensive evaluation of the patient’s presenting symptoms, vital signs, physical examination, 12-lead electrocardiography, and prompt device interrogation is all part of the first assessment.^76^ An emergent bedside echocardiogram is necessary to evaluate lead placement and rule out tamponade when hemodynamic instability is present^77^ (shown in Figure 3).

Clinical and imaging-based risk stratification aids in determining the urgency of further testing. High-risk patients need immediate imaging and may need surgery if they have unstable hemodynamics, significant pericardial effusions, or worsening symptoms. In contrast, low-risk individuals who have steady parameters and just slight deviations can be assessed less urgently.^78^^,^^79^

TTE is the first imaging modality used on stable patients. Noncontrast cardiac CT should be performed to confirm the diagnosis and direct treatment if the results are unclear, but suspicion is still high.^37^^,^^80^^,^^81^ Unless rapid vision is required to guide urgent treatment, conclusive imaging should be delayed until stabilization in unstable patients.^82^ Given that each modality has limits and a multimodal approach often produces the most accurate findings, the final diagnosis should ultimately include clinical context, device data, and imaging evidence.^77^^,^^83^

## Management strategies

The timing of the rupture, the severity of the symptoms, the hemodynamic stability, and the institutional knowledge all influence the therapy of cardiac lead perforation, necessitating a patient-specific strategy.^61^ The spectrum of treatment includes anything from surgical intervention in life-threatening situations to conservative observation in clinically stable persons.^41^ Treatment routes must be guided by proper risk assessment and accurate diagnosis.

### Conservative management

For individuals who are asymptomatic or just slightly symptomatic, conservative therapy is a good choice, especially when a delayed perforation is unintentionally found during regular follow-up. Patients who present more than a month after device implantation, have stable electrical parameters on device interrogation, and do not have a large pericardial effusion are the best prospects. The perforating lead may remain functionally quiescent in these individuals if there are no increasing sensing or pacing problems.^10^

Conservative management relies heavily on monitoring procedures. To identify arrhythmias or hemodynamic changes in real time, continuous telemetry is advised throughout the first observation period.^21^ Initially, serial device interrogations should be conducted every 6–12 hours to check for electrical degradation, such as changed sensitivity or increased capture thresholds. To check for new or expanding pericardial effusions, TTE should be performed every 24–48 hours, particularly during the first 48–72 hours after diagnosis.^84^

Modification of activity is also crucial. To reduce mechanical displacement of the lead, patients should briefly limit movement of the upper extremities on the implantation side.^21^ Because they might worsen lead migration or cause pericardial hemorrhage, Valsalva maneuvers and demanding exercises that increase intrathoracic pressure should be avoided.^85^

Reprogramming the device could be required to maximize performance and minimize any possible issues. Modifications include raising the pacing power to guarantee steady myocardial capture and altering the sensing thresholds to prevent unwarranted extracardiac signal detection.^86^ It can be necessary to temporarily disable rate-responsive characteristics in situations involving extracardiac stimulation, such as diaphragmatic pacing.

In 60%–85% of instances with stable, long-lasting perforations that do not exhibit severe electrical degradation or effusion, conservative measures may be effective.^10^ However, conservative treatment is often less successful in cases with acute or subacute perforations, particularly when symptoms change or electrical characteristics start to deteriorate. To prevent delayed escalation in care, timely re-evaluation is essential.^40^

### Lead repositioning and extraction

Lead repositioning or extraction is the last treatment for individuals who are hemodynamically unstable, exhibiting escalating electrical abnormalities, or symptomatic,^87^^,^^88^ although according to Younis et al^89^ in their cohort of 38 patients, 72% of whom had iatrogenic perforated pacemaker leads in the RV, simple traction with or without a locking stylet was achieved in all cases with emphasis that, given the possible situational consequences, surgical backup should be present at all times; these findings support the safety of nonsurgical management of lead perforations. If there is little fibrosis and no indication of major problems, simple fluoroscopic retraction may be tried in situations involving leads that were inserted for less than 6 weeks.^87^ When the etiology of initial perforation is high apical pressure, leads may be moved to more secure cardiac regions, such as the IVS, instead of the apex.^88^^,^^90^ In these situations, passive-fixation leads could be better because they lower the chance of recurrent perforation.^91^

Different percutaneous extraction methods are used depending on the fibrotic load and lead age.^87^^,^^91^ Manual traction or stylet-based removal is often enough for freshly inserted leads.^87^ Securing the lead and making withdrawal easier via thick adhesions, locking stylets, and telescoping sheaths increase success for leads that have been in place for many months.^87^^,^^91^ Energy-based instruments such as laser sheaths or radiofrequency-powered devices could be required for leads with implantation times longer than a year to safely break up fibrosis and allow extraction.^87^^,^^91^

Safety throughout procedures is crucial.^87^^,^^91^ Throughout the intervention, hemodynamic monitoring should be continued, and echocardiography should be accessible right away to check for the formation of pericardial effusions.^88^^,^^90^ Equipment for pericardiocentesis should be at the bedside, and cardiac surgery support is crucial, especially in high-risk situations.^87^^,^^88^^,^^91^

Patient stability determines when to intervene.^87^^,^^88^ Patients with active tamponade or those who are hemodynamically unstable need to be treated right away.^87^^,^^88^ In contrast, lead extraction should be done within 24 hours for stable individuals who exhibit symptoms of a perforation.^87^^,^^88^ If the risk-benefit analysis favors intervention, chronic, asymptomatic perforations may be treated electively.^87^^,^^88^^,^^91^

With success rates of 90%–95%, lead repositioning and extraction provide very positive results in centers with expertise.^87^^,^^91^ Approximately 1%–3% of instances result in major consequences such as hemothorax, vascular damage, or cardiac tamponade. Percutaneous extraction-related mortality is uncommon, estimated to be between 0.1% and 0.3%, especially when suitable surgical support is present.^87^^,^^91^

Transvenous lead extraction in confirmed lead perforation cases seems to have high procedural success and reasonable safety. In a retrospective cohort, all 17 perforating leads undergoing transvenous lead extraction were removed successfully without procedure-related mortality or major adverse events.^4^ However, data on pericardial effusion and tamponade during extraction are limited. Among extraction cohorts, the incidence of new-onset pericardial effusion is approximately 6.6%, with severe effusion in 3.3% and some requiring pericardiocentesis or surgery.^92^ These findings suggest that, even in confirmed perforation, extraction is feasible but carries an elevated risk of effusion and tamponade, warranting readiness for prompt pericardial drainage and surgical backup.

### Surgical management

**Figure 4:**
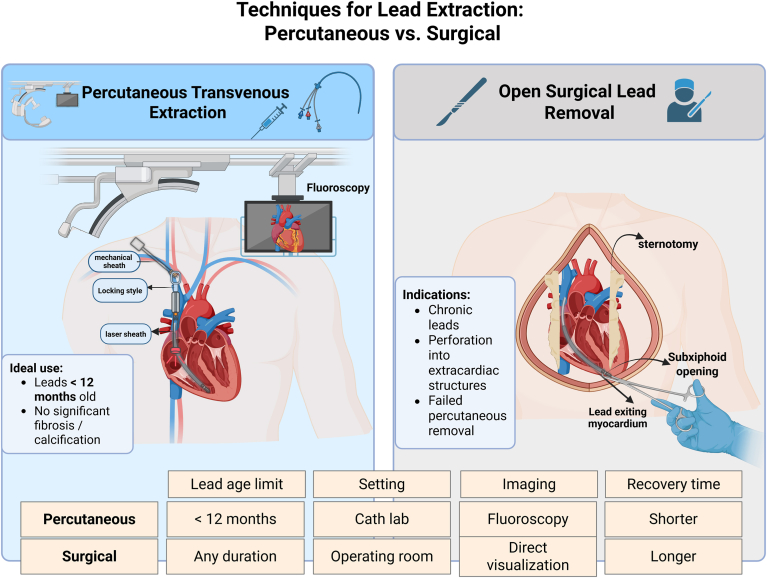
Side-by-side comparison of percutaneous transvenous and open surgical techniques for cardiac lead extraction. Method choice is shaped by lead dwell time, fibrotic burden, and imaging requirements. Percutaneous extraction relies on venous access and continuous fluoroscopy, making it ideal for relatively new leads with minimal scarring, whereas open surgical removal offers direct visualization and is reserved for chronic, fibrotic, perforated, or otherwise complicated cases. ICD = implantable cardioverter-defibrillator; RV = right ventricle.

When percutaneous approaches are not appropriate or have not worked or when consequences necessitate direct repair, surgical care is reserved for certain situations. Ineffective percutaneous extraction; extensive or loculated pericardial effusions that are not amenable to drainage; damage to nearby thoracic organs, including the lung or major arteries; complicated perforations involving several lines; and thick fibrosis are among the indications for surgery^71^^,^^93^, ^94^, ^95^, ^96^, ^97^ (shown in Figure 4).

The intricacy of the situation determines which surgical method is used.^71^^,^^94^^,^^95^^,^^97^ For isolated lead removal without further cardiac pathology, less invasive techniques such as thoracoscopic extraction or subxiphoid pericardial windows may be adequate.^91^^,^^95^ Full median sternotomy is still the gold standard for more severe injuries or situations that need concurrent cardiac treatments (eg, valve repair).^94^^,^^95^

Being prepared for temporary pacing is an essential part of perioperative treatment, particularly for patients who are pacemaker dependent.^71^^,^^94^^,^^97^ The necessity for thromboprophylaxis and hemostasis must be balanced while managing anticoagulation status, especially in patients with mechanical valves or significant thromboembolic risk.^71^^,^^94^ Depending on the new lead design and cardiac performance, postoperative device reprogramming should be customized.^91^

Excellent surgical results are obtained; in more than 99% of instances, a successful extraction is accomplished.^97^ Depending on the intricacy of the procedure and the patient’s level of recuperation, the average length of stay in the hospital is 3–7 days. Compared with sternotomy (8–12 weeks), minimally invasive procedures need less recovery time (4–6 weeks), and most patients resume their normal activity levels within this period.^94^^,^^95^

### Special management considerations

Modified management methods are required in a number of therapeutic situations. To restore hemodynamic stability in tamponade instances, prompt echocardiography-guided pericardiocentesis is necessary. If the effusion returns, it could be necessary to place an indwelling pericardial drain, and depending on the risk of bleeding, anticoagulation should be reversed.^98^

Extraction has to be done very carefully when extracardiac tissues are implicated, such as pulmonary or vascular perforation. To assure safety, a multidisciplinary team including interventional radiology or thoracic surgery may be required, along with modified procedures.^98^

Planning for temporary pacing during lead removal is crucial for individuals who are pacemaker dependent.^52^^,^^35^ Depending on patient anatomy and urgency, this may include starting transcutaneous pacing or putting a temporary lead on the contralateral side. To avoid bradyarrhythmic problems, reimplantation of a new lead is sometimes done during the same surgery for these individuals.^35^

Recent studies have explored the safety profile of left bundle branch area (LBBa) pacing, particularly with regard to myocardial perforation risk. Although LBBa pacing involves deeper septal lead placement than traditional RV pacing, the incidence of perforation seems low, ranging from 0.2% to 0.8% in large series, as evidenced in studies such as those of Huang et al^68^ and Vijayaraman et al.^99^ The use of fixed-curve sheaths (eg, C315 His or Select Site C304) combined with electroanatomic and fluoroscopic guidance facilitates precise lead positioning and may reduce perforation risk by stabilizing engagement and limiting free-wall advancement. Some studies suggest that the fibrotic nature of the IVS, along with continuous pacing impedance and electrocardiographic monitoring during lead deployment, adds additional safety compared with apical or free-wall lead placements. Nonetheless, septal hematoma, delayed perforation, and coronary artery injury remain potential complications, especially in patients with thin septa or on anticoagulation.^100^

The mechanical and anatomic risk factors that caused the original hole must be considered in reimplantation techniques. Passive attachment techniques and placement in the septal wall rather than the apex are often recommended.^35^ Device-dependent patients often need same-day reimplantation, whereas other patients may be planned for a delayed approach. Reimplantation timing is contingent upon stability and risk.^35^

## Prevention strategies and risk mitigation

From patient selection to long-term postimplantation monitoring, preventing cardiac lead perforation is an essential part of managing CIEDs.^101^ Vigilant follow-up, procedural optimization, and risk stratification are all components of a complete preventative approach.^101^^,^^102^

### Preimplantation risk assessment

To reduce the risk of cardiac perforation during CIED operations, a thorough preimplantation risk assessment is essential. The clinical justification for device treatment and individual risk factors for consequences, including perforation, should be carefully evaluated before choosing a patient, according to the medical literature.^7^^,^^21^^,^^103^ Advanced age, female sex, low BMI, long-term corticosteroid treatment, and active-fixation lead consumption are key risk factors that have been repeatedly established.^36^^,^^104^ These elements are linked to heightened tissue brittleness and an elevated risk of cardiac damage with the lead implantation.

Using clinical and demographic factors such as those mentioned earlier, risk stratification techniques and scoring systems have been created to help identify individuals who are at a higher risk of perforation.^36^^,^^104^ Even though these technologies have potential, particularly when combined with machine learning techniques and big clinical datasets, they need further prospective validation before being widely used in clinical settings.^103^^,^^104^

Alternative therapy approaches should be taken into consideration for patients who have been classified as high risk. Pharmacologic treatment may be enough in some bradyarrhythmia instances, negating the requirement for device placement.^21^ Subcutaneous ICDs lower the risk of cardiac perforation in patients who need defibrillation but not pacing because they do not need transvenous connections.^105^ Given that leadless pacemakers completely eliminate the need for transvenous access, they are an appealing alternative for single-chamber ventricular pacing. However, it should be noted that although leadless systems lower the risk of infection, they may be slightly more susceptible to perforation than traditional transvenous pacemakers, especially in some populations.^105^

Although leadless pacemakers eliminate transvenous leads, cardiac perforation remains a recognized risk, often involving the inferior or apical RV wall. Real-world data show slightly higher rates of early perforation/pericardial effusion with leadless systems (∼0.8%) than transvenous pacemakers (∼0.4%) in the first 30 days.^106^ Despite this, leadless devices are associated with fewer overall complications, fewer reinterventions, and lower chronic complication rates over longer-term follow-up.^107^ Patients who experience perforation with these leadless devices may face more severe clinical outcomes, sometimes requiring urgent pericardiocentesis or surgical intervention.^107^^,^^108^ These findings suggest that although leadless pacing reduces lead-related issues, operator technique is critical, especially regarding implantation site selection to minimize RV wall injury.

Along with preoperative imaging and laboratory examination where necessary, preprocedural planning should also include thorough collaborative decision making that considers the patient’s comorbidities, frailty, and preferences.^103^ To reduce the risk of perforation, procedural considerations are also crucial. These include the kind of lead used (active-fixation leads have a greater risk of perforation) and the intraoperative evaluation of lead position and slack.^36^^,^^104^

In conclusion, current guidelines and recent clinical studies support the best practices for preventing perforations during CIED implantation: careful procedural technique, careful patient selection, individualized risk assessment, and consideration of alternative device strategies in high-risk patients.^36^^,^^104^^,^^105^

### Procedural modifications

The risk of perforation during the surgery may be considerably decreased by making adjustments to the implantation method and the choice of device.^35^^,^^63^^,^^109^ Selecting the right lead is essential; if the anatomy allows for sufficient contact and stability, passive-fixation leads may be a better option for high-risk patients.^35^^,^^37^ Selecting leads with a shorter helix, steroid-eluting tips, and less stiffness may lessen the likelihood of perforation when active fixation is required.

Lead stiffness during implantation is affected by the stylet type and how far it is inserted. Bench testing shows that firmer stylets increase tip pressure, which may raise the risk of myocardial perforation, especially in thin septal areas. In contrast, soft or extra-soft stylets reduce tip force, potentially improving safety but may offer less control during implantation.^110^ Studies also show that stylet-driven leads (which rely on internal stylets) tend to be stiffer and faster to deploy, but may carry a slightly higher risk of complications such as dislodgement, although not necessarily perforation.^111^ Therefore, stylet selection and positioning should be adjusted carefully to balance control with safety in procedures such as LBBa pacing.

Procedural safety is also affected by lead handling and access site selection.^37^ Compared with conventional subclavian puncture, axillary venous access is linked to a more favorable lead trajectory and may result in less mechanical stress.^112^ To guarantee proper lead insertion and prevent unintentional progression beyond the cardiac outline, operators should use various fluoroscopic views. Myocardial mechanical damage may be reduced with careful lead manipulation that uses mild advancement and a suitable stylet shape.^35^^,^^109^^,^^112^

The risk of perforation is significantly influenced by the anatomic location of lead implantation. Despite its widespread usage, the RV apex is linked to a weaker myocardial wall and a higher risk of penetration. Other locations with thicker walls, including the low septum, the RV outflow tract septum, or the midseptum close to the moderator band, could provide more stable fixation with a decreased risk of perforation.^13^

To guarantee mechanical integrity and electrical function, intraoperative testing is necessary. Early identification of unstable lead placements is made possible by real-time evaluation of sensing, impedance, and capture thresholds. Intermittent instability may be detected by provocative testing in various body postures, and diaphragmatic or extracardiac stimulation, which may suggest that the lead tip is close to or beyond the myocardial boundary, can be revealed with high-output pacing (eg, at 10 V).^57^

### Postimplantation management

The goal of postimplantation interventions is to lower the risk of negative outcomes and identify lead-related problems early.^10^^,^^113^^,^^114^

Without using too high voltage outputs that would cause progressive lead migration, device programming must be tuned to enable consistent myocardial capture with a sufficient safety margin. Inappropriate device behaviors resulting from partial or intermittent lead perforation may be avoided by modifying sensing vectors and thresholds.^10^^,^^113^

To track lead function over time, systematic follow-up is essential.^87^ It is advised to do an early clinical assessment 24–72 hours after implantation to detect any acute problems.^115^ Lead stability and function may be continuously monitored with follow-up assessments at 2–4 weeks, 3 months, and thereafter at regular intervals (usually every 6–12 months).^87^

The capacity to identify early indicators of lead-related problems has greatly increased, thanks to remote monitoring systems. These devices can identify small changes in lead performance that may occur before clinical symptoms appear, such as increasing capture thresholds, lowering impedance, or aberrant sensing. To improve early detection and reduce false alarms, customized alert levels have to be set.^114^

Finally, patient education is a critical component of prevention. The warning signals of a lead perforation, such as dizziness, syncope, persistent hiccups, chest discomfort, or dyspnea, should be explained in detail to patients. Both written and spoken educational resources should support this knowledge and include instructions on when to seek emergency medical care. Giving people the tools to identify and report any issues is a simple but very powerful way to speed up diagnosis and enhance results.^10^^,^^113^

## Special populations

**Figure 5:**
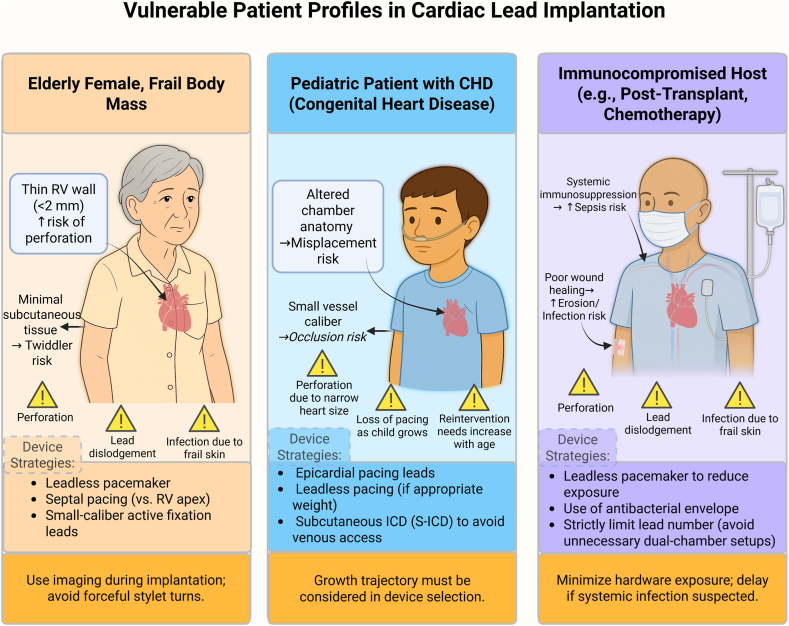
Illustrated overview of 3 high-risk populations in whom standard transvenous lead placement may pose elevated risks. Elderly patients with low body mass index have anatomic frailty, pediatric patients with congenital heart disease require growth- and anatomy-sensitive planning, and immunocompromised patients are prone to infection and tissue breakdown. Recommended strategies include leadless pacing, epicardial leads, and minimal-hardware configurations.

Certain populations must be managed cautiously owing to the risk of various complications such as device infection, which is estimated to occur at a rate of 0.5% after primary implantation and up to 7% after secondary manipulations,^116^ which can even be complicated by endocarditis, which is an indication for removal^85^ (shown in Figure 5).

Therefore, leadless pacemakers emerge as a solution to the previously mentioned issue given that it has been shown to decrease the risk of such complications through encapsulation of the device; in addition, the position and the need to keep a foreign object in the venous circulation also add additional protective layers against infections.^117^, ^118^, ^119^ El-Chami et al^119^ state that, because leadless pacemakers have a small surface area, they convey a less theoretical risk of infection.

## Emerging technologies and future directions

Conventional cardiac pacemakers pose a lot of risks to patients, considering the rates of complications associated with the traditional design, including mechanical, infectious, and device-related issues.^120^

As mentioned before, leadless pacemakers are promising in the world of cardiac pacing. As the field is rapidly advancing, we are seeing a potential for a future where leadless pacemakers are the first-line solution for patients in need, especially with the newly developed systems that can provide any type of pacing without the need for transvenous leads.^121^, ^122^, ^123^

With that said, complex pacing modalities, device interactions, longevity differences in multiple devices, and the missing developed techniques in transvenous pacing, which leadless pacemakers need to reach the level of, still require further research to understand and solve.

### Guidelines and recommendations

A direct clear management guidelines in cardiac lead perforations is still needed to be developed, although many studies have been conducted; for instance, Rajkumar et al^4^ showed that CT imaging was attributed to be the imaging modality of choice when it comes to cardiac perforations, which was consistent with that of Zhou et al^52^ in which transvenous lead extraction was used and showed safe and efficient in managing cardiac lead perforations.

Chest CT is widely used for detecting lead perforation owing to its availability and high resolution. However, metallic leads often cause blooming and beam-hardening artifacts, which may mimic lead extension beyond the myocardium and result in false-positive diagnoses of perforation. To avoid overinterpretation, CT findings should be correlated with clinical context and, where possible, confirmed by echocardiography or fluoroscopic imaging.^124^^,^^125^

Recently, Kidoh et al^125^ tested a newly proposed imaging modality that uses the metal artifact reduction algorithm to lower diagnostic uncertainty and improve clinical management and showed that, in 47 consecutive cases, the metal artifact reduction algorithm was able to reduce metal artifacts and improve the produced images, as opposed to conventional CT where in a quarter of cases the results were equivocal and nonconclusive.

Given that no evidence-based management options exist for cardiac lead perforation, Rav Acha et al,^10^ in their retrospective study on 48 perforation cases, 22 of which were associated with recurrent symptoms, concluded that lead revision is a safer and more effective option in cardiac lead perforations, especially in patients on blood thinners (anticoagulants and antiplatelets). Therefore, we recommend that a consensus and clear guideline be sought and developed to lower the risk of devastating complications and improve patient care.

## Conclusion and future research priorities

If not detected and treated right away, cardiac lead perforation, an uncommon but dangerous side effect of CIEDs, could be fatal. In addition to developments in diagnostic methods and therapy options ranging from conservative surveillance to surgical intervention, current research has greatly increased our awareness of the anatomic, procedural, and patient-specific risk factors involved.

Several significant gaps still exist despite these advancements. First, given that some instances are silent and diagnostic criteria are unclear, the real frequency of subclinical and delayed perforations is still underestimated. Furthermore, there is a dearth of prospective, high-quality data on the long-term results of conservatively handled patients as opposed to those who have extraction or relocation. Device-specific risk profiles are continually developing and should be continuously monitored and assessed, particularly for more recent technologies such as subcutaneous ICDs and leadless pacemakers.

There is an urgent need to standardize reporting and diagnostic criteria. Different definitions of lead perforation, such as those based on electrical characteristics, imaging thresholds, or clinical presentation, make it difficult to compare research and reduce the generalizability of results. The quality and comparability of future research will be improved by establishing consistent categorization schemes and core result sets.

Prospective multicenter registries to capture actual incidence and outcomes; machine learning-based risk stratification tools that integrate imaging, clinical, and procedural variables; randomized controlled trials assessing the best management practices for asymptomatic or minimally symptomatic perforations; and design optimization and postmarketing surveillance for leadless and alternative pacing systems are examples of high-priority areas for future scholarly research.^85^

Finally, it is critical to promote international cooperation via research networks and shared datasets. These initiatives may assist in tailoring risk-reduction plans for lead implantation, expediting the gathering of evidence, and influencing the creation of guidelines. The creation of surveillance registries that monitor perforation incidence, intervention results, and long-term device safety profiles, as well as an international agreement on best practices, would be very beneficial to the field.
